# Metalloproteinases in Cardiac Surgery: A Systematic Review

**DOI:** 10.3390/biom13010113

**Published:** 2023-01-05

**Authors:** Giuseppe Filiberto Serraino, Federica Jiritano, Davide Costa, Nicola Ielapi, Domenica Battaglia, Umberto Marcello Bracale, Pasquale Mastroroberto, Michele Andreucci, Raffaele Serra

**Affiliations:** 1 Department of Experimental and Clinical Medicine, University “Magna Graecia” of Catanzaro, 88100 Catanzaro, Italy; 2 Department of Law, Economics and Sociology, University “Magna Graecia” of Catanzaro, 88100 Catanzaro, Italy; 3 Interuniversity Center of Phlebolymphology (CIFL), International Research and Educational Program in Clinical and Experimental Biotechnology, Department of Surgical and Medical Sciences, University “Magna Graecia” of Catanzaro, Viale Europa, 88100 Catanzaro, Italy; 4 Department of Public Health and Infectious Disease, “Sapienza” University of Rome, 00185 Roma, Italy; 5 Department of Public Health, University Federico II of Naples, 80138 Naples, Italy; 6 Department of Health Sciences, University “Magna Graecia” of Catanzaro, 88100 Catanzaro, Italy; 7 Department of Medical and Surgical Sciences, University “Magna Graecia” of Catanzaro, 88100 Catanzaro, Italy

**Keywords:** matrix metalloproteinases, cardiopulmonary bypass, cardiac surgery, inflammation, SIRS

## Abstract

The role of matrix metalloproteinases (MMPs) in routine cardiac operations including cardiopulmonary bypass (CPB) is still poorly explored. The purpose of this systematic review was to thoroughly summarize and discuss the existing knowledge of the MMP profile in cardiac surgery. All studies meeting the inclusion criteria (i.e., those reporting detailed data about MMP release during and after CPB) were selected after screening the literature published between July 1975 and August 2022. Fifteen trials that enrolled a total of 431 participants were included. MMP levels were found to be significantly correlated with CPB in all included studies. The gelatinases MMP-2 and MMP-9 were highly released in cardiac surgery with CPB. MMP-9 levels were found to be increased after CPB start and during the duration of CPB. Particularly, it is overexpressed both in the myocardial tissue and circulating in the bloodstream. Also, MMP-2 levels increased after CPB both in plasma and in myocardial tissue. MMP-7, MMP-8, and MMP-13 levels increased after CPB start and remained elevated up to 6 h later. Increased levels of MMPs were associated with adverse post-operative outcomes. Conversely, TIMP-1 decreased with CPB. Mechanical and pharmacological strategies were applied in two studies to analyze their effect on the inflammatory response to cardiac surgery and CPB and on postoperative outcomes. New targeted MMP inhibitor therapies could protect against systemic inflammatory response syndrome after CPB and should be the subject of future large prospective multicenter randomized clinical trials.

## 1. Introduction

Cardiac surgery with cardiopulmonary bypass (CPB) is often associated with systemic inflammatory response syndrome (SIRS), significantly affecting postoperative mortality and morbidity [1]. SIRS may result from surgical trauma, endotoxemia, ischemia-reperfusion damage, or interaction between blood components and the artificial surface of the bypass circuit [1]. This inflammatory response may have an impact on the onset of postoperative issues such as cardiac dysfunction, respiratory failure, renal and neurologic dysfunction, bleeding disorders, altered liver function, and ultimately multiple organ failure (MOF) [2]. Multiple pro-inflammatory cytokines are released as a result of the activation of the immune system, leucocytes, and endothelial cells, as well as the complement system [2]. During cardiovascular surgery with CPB, the synthesis of cytokines such interleukin (IL), tumor necrosis factor (TNF), kallikrein, and bradykinin increases, which exacerbates the inflammatory response [2].

The matrix metalloproteinases (MMPs) are a group of zinc- and calcium-dependent endopeptidases that are involved in the degradation of connective tissue’s extracellular matrix [3]. These proteinases are crucial for both pathologic (such as cancer) and physiological (such as growth and development) activities [3]. A few MMP species have also been linked to cardiovascular remodeling processes like aneurysm and atherosclerotic plaque development [4]. The MMP enzyme system has been shown to be quickly activated in inflammatory processes as well as in acute myocardial infarction [5]. After CPB, bioactive peptides can cause the release and activation of MMPs [6]. It has been demonstrated that a number of cytokines increase MMP expression [7,8]. The role of MMPs in the cardiovascular system is less well explored. It is even less known whether or which MMPs can be released following routine cardiac operations, including CPB. Determining if and which MMPs are released into the systemic circulation during and after CPB was the aim of the current systematic review. Additionally, any methods for reducing MMP production were also mentioned.

## 2. Materials and Methods

### 2.1. Protocol

The review was performed in accordance with instructions given by the Cochrane Handbook for Systematic Reviews of Interventions [8]. The systematic review was performed according to PRISMA (Preferred Reporting Items for Systematic Reviews and Meta-Analyses) guidelines. The search methods, data extraction, assessment, and presentation were performed as recommended by the Cochrane Handbook for Systematic Reviews of Interventions (Version 5.1).

### 2.2. Eligibility Criteria

Randomized (RCTs) and non-randomized controlled trials (NRCTs), as well as prospective and retrospective observational cohort studies, irrespective of blinding, language, publication status, and date of publication, were considered eligible for this study. Participants of any age undergoing cardiac surgery with cardiopulmonary bypass were considered. Studies were not included in the analysis if they met one of the following exclusion criteria: (i) the analysis was a review, case report, case series (<10 patients), or a conference abstract; or (ii) the analysis provided incomplete information about study objectives. Pre-clinical studies (non-human studies) were excluded from the main analysis. Inclusion and exclusion criteria for qualitative/quantitative analyses were summarized according to the PICo (population/patient/problem, interest, context) approach (Table S1 of Supplementary Material).

### 2.3. Subject of Interest

We assessed trials evaluating the role of metalloproteinases in subjects undergoing cardiac surgical operations with cardiopulmonary bypass.

### 2.4. Information Sources

Potentially eligible studies were identified after an extensive search of the literature conducted through PubMed and Scopus without date or language restrictions. Keywords and MeSH terms pertinent to the exposure of interest were used in relevant combinations: “metalloproteinases”, “metalloproteases”, “TIMP”, “cardiopulmonary bypass”, and “cardiac surgery”. The literature search was run to identify studies published between July 1975 and August 2022. In addition, we searched trial registries, and reference lists were carefully analyzed for pertinent studies. Case reports, opinions, and editorials were excluded.

### 2.5. Study Selection and Data Items

Two reviewers (F.J., G.F.S.) identified trials for inclusion independently of each other. Excluded studies and reasons for exclusion were recorded. Two authors independently screened the search output to identify records of potentially eligible trials examining outcomes, the full texts of which were retrieved and assessed for inclusion. A standardized form was used to extract data from the included studies for the assessment of study quality and evidence synthesis. Extracted information included the following: year of publication; study population, with inclusion and exclusion criteria; sample size; participant characteristics; baseline characteristics; outcomes; and information for the risk of bias assessment. Data extraction forms were completed by one author and checked by a second author. Likewise, quality assessment was performed by one author and checked by a second.

### 2.6. Risk of Bias in Individual Studies

The methodological quality of randomized trials was assessed using the Cochrane Collaboration’s tools for assessing risk of bias in parallel group and cluster randomized trials [9,10]. The items assessed for parallel group trials were as follows: (i) sequence generation; (ii) allocation concealment; (iii) blinding of outcome assessor; (iv) incomplete outcome data; (v) selective outcome reporting; and (vi) other sources of bias, including funder bias. Risk of bias was graded as unclear, high, or low. We graded sealed opaque envelopes as unclear evidence of allocation concealment. We also considered the absence of a prespecified protocol or trial registration of the trial design as unclear evidence of reporting bias. The risk of bias items assessed for cluster randomized trials were as follows: (i) recruitment bias; (ii) baseline imbalance; (iii) loss of clusters, incorrect analysis; and (iv) comparability with individually randomized trials.

For NRCTs, a modified Newcastle–Ottawa quality assessment scale for cross-sectional studies was used to assess the quality of the included studies. The total score for the modified Newcastle–Ottawa scale for cross-sectional studies is nine (9) stars as a maximum for the overall scale, with a minimum of zero. A study was considered high quality if it achieved 7 out of 9 and medium quality if it achieved 5 out of 9 (Table S2 of Supplementary Material). Overall quality was independently determined by each reviewer, with discrepancies resolved by consensus. 

## 3. Results

A total of 142 abstracts were retrieved from the searches (Figure S1 of Supplementary Material). There were 142 articles screened and 122 excluded. A total of 20 relevant publications were retrieved for further assessment. Fifteen trials that enrolled a total of 431 participants met the inclusion criteria and were included in the systematic review [6,7,11,12,13,14,15,16,17,18,19,20,21,22,23]. Two review authors (F.J., G.F.S.) agreed on the selection of included studies. Key characteristics of individual studies are described in Table 1.

### 3.1. Included Studies

Of the 15 studies included, 5 were prospective RCTs (*n* = 211) [6,7,16,18,19], and 1 was a retrospective observational study [23], whereas the remaining 9 were prospective observational NRCTs [11,12,13,14,15,17,20,21,22]. All studies included patients submitted to cardiac surgery with CPB, but some better defined their population: seven studies enrolled patients undergoing CABG with CPB (*n* = 213) [7,11,12,13,14,16,21], one study included patients submitted to off-pump or on-pump CABG [15], one enrolled patients submitted to pericardiectomy with CPB [23], and one included patients undergoing to CABG or valve replacement with CPB [6].

MMP-9 was the metalloproteinase investigated in all trials. Seven trials studied MMP-2 [6,7,11,12,14,19,23], and three papers studied MMP-8 [7,12,21]. MMP-1 was also studied in one trial [23], MMP-3 was investigated in one paper [21], and MMP-7 was analyzed in one trial [17], while MMP-13 was evaluated in one study [12]. Five articles studied TIMP-1 [13,14,15,16,23], while just one paper evaluated TIMP-4 [11].

### 3.2. Excluded Studies

Five trials that met our inclusion criteria were excluded after review of the full manuscript (Table S3 of Supplementary Material). Four trials were excluded because they were animal research [24,25,26,27]. One trial was excluded because it was an in vitro study and the role of CPB was not investigated [28].

### 3.3. Main Findings

The MMPs analyzed in cardiac surgery patients with CPB are described in Table 2.

MMP levels were found to be significantly correlated with CPB in all included studies. MMP-9 levels were found to be increased after CPB start and during CPB [6,7,11,12,13,14,15,16,17,18,19,20,21,22,23]. It is particularly overexpressed both in the myocardial tissue and circulating in the bloodstream [11,14]. MMP-9 levels rose especially after cross-clamp removal and for 6 h after CPB [7,12,14,16,18,20]. In addition, MMP-2 levels increased after CPB both in plasma and in myocardial tissue [11,14]. Conversely, TIMP-1 decreased with CPB [13,14].

Mechanical and pharmacological strategies were applied in two studies to analyze their effect on the inflammatory response to the cardiac surgery and CPB and on postoperative outcomes [6,16,18,21]. 

### 3.4. Risk of Bias and Study Quality

A summary of the risk of biases of the included trials is reported in the Supplemental Data (Figures S2 and S3). Quality assessment for observational studies showed no high-quality studies, two medium-quality studies [15,21,22], and seven low-quality studies. Four out of five trials showed unclear random sequence generation. All showed adequate completeness of outcome data and unclear risk of reporting bias. Only one trial disclosed funding sources [7]. Unclear evidence of blinding of outcome assessors and detection bias was found in two studies [6,7,16]. Three trials had low risk for allocation concealment [6,16,18] and the other two trials had unclear risk [7,19]. The individual bias domains are presented in the risk of bias Supplementary Material.

## 4. Discussion

To the best of the authors’ knowledge, this systematic review is the first to provide an in-depth description of the role of metalloproteinases in patients undergoing cardiac surgery with CPB.

CPB is still essential for carrying out cardiac surgeries [29]. However, a wide range of immunological and physiological alterations can be linked to CPB [1,2]. An inflammatory reaction takes place, which sporadically results in the emergence of secondary organ dysfunction, including lung or kidney damage [1,2]. Systemic inflammatory response syndrome includes the activation of neutrophils, which may release a variety of reactive oxygen species and proteolytic enzymes, including MMPs [15]. Inflammation requires leukocyte infiltration into tissue, and MMPs support leukocyte extravasation and infiltration [30]. MMPs are a family of more than 25 species of calcium- and zinc-dependent proteases that are synthesized as inactive zymogens (pro-MMPs) [31]. They are essential for normal tissue remodeling [31]. MMPs are the predominant proteases responsible for degradation of extracellular matrix proteins, and are thus thought to play a key role in angiogenesis, inflammatory processes, cancer development, cell proliferation, and apoptosis [31]. The activity of MMPs is regulated at the level of transcription, activation, and inhibition by tissue inhibitors of metalloproteinases (TIMPs) [32]. MMPs are either integrated into the membrane as fully active enzymes or are generated as inactive zymogens that are released into the extracellular space as pro-enzymes [31].

MMPs are classified into three groups: interstitial collagenases, gelatinases, and stromelysins. Among the interstitial collagenases, MMP-1, MMP-8, and MMP-13 were investigated in cardiac surgery patients with CPB.

MMP-1 is the most common interstitial collagenase. Types I, II, and III of fibrillar collagen as well as a few additional extracellular matrix proteins are cleaved by MMP-1 [33]. Since type I and type III collagens constitute 90% of the protein in the cardiac extracellular matrix, collagenases are crucial for the turnover of the extracellular matrix in the myocardium [34]. MMP-8 (neutrophil collagenase or collagenase-2) can instead promote the degradation of the fibrillar collagens such as collagen type I, which is a major component of atherosclerotic plaques [33,35]. Additionally, MMP-8 can process a variety of non-collagenous substances, contributing to immunological responses [33,35]. As with the other collagenases, MMP-13 (collagenase-3) degrades casein, collagen, fibrinogen, and gelatin. Cardiovascular fibroblasts and macrophages both express MMP-13. Its increased expression is linked to a rise in collagenolysis in atheromatous plaques, indicating that MMP-13 promotes instability in the plaques [36].

MMP-2 and MMP-9 are two gelatinases described in patients submitted to CPB. Cardiomyocytes, fibroblasts, endothelial cells, and inflammatory cells can all generate and secrete MMP-2, also known as gelatinase A, in the heart. Gelatin, fibronectin, and nonfibrillar collagens (such as collagen IV, V, VII, and X) are the main substances that MMP-2 cleaves. When exposed to angiotensin II or hypoxia, MMP-2 activity is decreased. MMP-2 activity is also controlled by cytokines, growth hormones, reactive oxygen species, and collagen. MMP-2 can degrade components of the contractile apparatus, such as troponin I and light chain myosin 1 [31,37]. MMP-9 (gelatinase B) and MMP-2 share a similar substrate profile. The transcriptional level of MMP-9 is regulated by cytokines and growth factors (IL-13, TNF, transforming growth factor beta (TGF), and vascular endothelial growth factor (VEGF)), as well as epigenetic mechanisms (histone modification, DNA methylation, and non-coding RNA). Collagen, fibronectin, and laminin are just a few of the extracellular matrix substrates that active MMP-9 enzymatically deteriorates to promote extracellular matrix turnover. Myocardial inflammation is reduced when MMP-9 expression or activity is inhibited, indicating that MMP-9 plays a significant role in unfavorable myocardial remodeling [31].

Additionally, the stromelysins MMP-3 and MMP-7 were also reported in cardiac surgery subjects that underwent CPB. Also known as stromelysin-1, MMP-3 is released by cardiac fibroblasts and macrophages. MMP-3 cleaves a variety of non-fibrillar collagens, laminins, gelatin, and fibronectin in addition to activating a number of other MMPs by zymogen cleavage. It is crucial in tissue remodeling by destroying extracellular matrix [31]. Considering that the MMP3 gene promoter shares structural similarities with the MMP1 and MMP9 promoters, it is not surprising that similar triggers stimulate MMP-3 expression. MMP-3-induced pathological alterations include endothelial damage and inflammatory cell activation [31]. Because MMP-3 can interact with plasminogen and fibrinogen, prolonged production of MMP-3 is closely associated with atherosclerotic plaque rupture [38]. In addition, MMP-7 is a key player in the destabilization of atheromatous plaques and has been connected to the apoptosis of vascular smooth muscle cells [39]. The fibrillar collagens I and III, the proteins of the basement membrane (collagen IV and fibronectin), and proteoglycans are all targets of MMP-7’s proteolytic action.

Because MMPs break down numerous constituents of the extracellular matrix (ECM), it is crucial to tightly regulate their activity in order to preserve the ECM’s regular composition and functionality. TIMP-1, -2, -3, and -4 are four endogenous proteins that tightly control the activity of MMPs [32]. TIMPs can bind to MMPs in a 1:1 ratio and form noncovalent complexes with them, which block the activity of all known MMPs [32]. Therefore, TIMPs are crucial in preserving the equilibrium between extracellular matrix remodeling and breakdown. TIMP-1 and TIMP-2 are multifunctional proteins with numerous functions that prevent angiogenesis [32]. TIMP-3 is solely present in the extracellular matrix [32]. It is regulated in a cell cycle-dependent mode and suppresses neovascularization [32]. TIMP-4 has been identified as the major MMP inhibitor in human platelets [32]. Under pathological conditions associated with unbalanced MMP activities, changes in TIMP levels are significant because they have a direct impact on MMP activity. However, the TIMP family can also control other important processes such as proliferation and apoptosis by mechanisms independent of their MMP inhibitory actions [32].

The inflammatory response to heart surgery and CPB was examined using these MMPs as biomarkers. In addition, several researchers tried to connect the variations in these levels to the organ dysfunction brought on by SIRS. First, Mayers and colleagues noted that following CPB, the release of MMPs and pro-MMPs in cardiac tissue increased [11]. They came to the conclusion that CPB caused acute inflammation and organ damage via increasing MMPs and decreasing TIMPs [11]. Similarly, other researchers described the release and synthesis of particular MMPs both before and after CPB [12,13,17,20]. In 2005, Lin and colleagues compared patients undergoing coronary artery bypass grafting (CABG) with CPB or with an off-pump approach in order to demonstrate the effect of CPB on MMP activity and release [15]. They found that the CPB group had higher plasma MMP-9 concentrations and activity as well as higher MMP-9/TIMP-1 ratios, which suggests that CPB may have a significant impact on the inflammatory response [15]. The myocardial activity of MMPs and cardiac function were initially found to be correlated by Lalu and colleagues [14]. According to their investigation, the early rise in MMP activity after CPB led to the proteolysis of troponin I or actin derangement, which may be a factor in myocardial stunning [14]. Furthermore, a high level of MMP-9 was linked to poor post-operative outcomes and was an independent risk factor for post-operative complications in 22 patients with constrictive pericarditis who underwent pericardiectomy with CPB [23]. Additionally, MMPs were discovered to be accurate biomarkers of acute kidney injury following CPB [22]. In 2008, a prospective randomized trial showed that aprotinin treatment decreased the blood levels of MMP-8 and MMP-9 during and six hours after CPB for the first time in humans [7]. Up until that point, only animal models had been employed to develop and successfully use pharmaceutical inhibitors of MMP activity [24]. Milrinone’s impact on CPB-related inflammation was examined by Gong and colleagues [18]. Milrinone patients showed a decreased release of MMP-9 [18]. Furthermore, employing MMPs as biomarkers, other researchers looked into a mechanical technique to contrast inflammation and lung injury [16,21]. They reached the conclusion that by maintaining ventilation during CPB, a lesser inflammatory and proteolytic response was produced, preserving pulmonary function [16,21]. To assess a cardioprotective effect, remote ischemic preconditioning (RIPC) episodes were examined [19]. After CPB in RIPC patients, decreased activity of MMP-2 and MMP-9 was discovered in cardiac tissue [19]. Additionally, there was a favorable correlation between serum troponin I concentrations and MMP levels, indicating the importance of MMPs in myocardial protection [19]. The study, however, was underpowered and had a number of drawbacks. Furthermore, Gao and colleagues recently investigated the role of doxycycline as an MMP inhibitor [6]. The doxycycline patients in their prospective RCT displayed decreased MMP-2 and MMP-9 concentrations following CPB [6]. The research group also had better early clinical outcomes [6]. Their findings supported prior animal model observations [26,27]. Given the small enrolled population, their results should be carefully considered.

### 4.1. Strengths and Limitations

This is the most comprehensive evaluation of MMPs in cardiac surgery using CPB to date. It employed extensive search techniques across many registries and data sources, had access to the full texts of all reported trials, used contemporary risk of bias assessments, and evaluated clinical outcomes. The quality and quantity of the available evidence is the primary limitation on the findings and interpretations of this systematic review. The review identified significant methodological issues in all of the research, which were limits of the existing data. The likelihood of procedural bias was considerable, and studies varied greatly in how results were reported.

### 4.2. Clinical Importance

It is essential to comprehend how MMPs function after cardiac surgery with CPB in order to create therapeutic approaches to prevent adverse clinical outcomes. Following a thorough investigation of their activity and release, all strategies to limit them became crucial for controlling the inflammatory response and lowering post-operative adverse outcomes. Unfortunately, implementing therapeutically useful treatments is challenging. The fact that the expression of these enzymes is not only affected by CPB usage but also by underlying heart disease presents difficulties in the development of selective metalloproteinase inhibitors. Additionally, numerous stimuli that work through a number of signaling pathways cause an increase in MMP expression, indicating that it may be more beneficial to modulate MMP “synthesis” as opposed to “activity”. Therefore, pharmacological approaches may specifically target a given stimulus (for example, cytokine receptor blockage) or a part of the intracellular signaling cascade it employs.

## 5. Conclusions

By understanding the molecular mechanisms regulating expression and activity of different MMPs during and after CPB, specific therapies that protect from systemic inflammation and consideration of the related clinical implications are needed. New targeted MMP inhibitor medicines could be the subject of large prospective multicenter RCTs.

## Figures and Tables

**Table 1:** Key characteristics of individual studies. Abbreviations: AKI, acute kidney injury; CABG, coronary artery bypass graft; CPB, cardiopulmonary bypass; IL, interleukin; LCN-2, lipocalin-2; MMP, metalloproteinase; NRCT, non-randomized controlled trial; NOS, nitric oxide synthase; RCT, randomized controlled trial; TIMP, tissue inhibitors of metalloproteinases; TNFα, tumor necrosis factor α; TxB2, thromboxane B2.

Study, Year [Ref]	Study Design	N° of Patients	Population	MMP	Other Factors	Outcomes	Main Findings
Mayers, 2001 [11]	Prospective observational NRCT	10	Patients undergoing elective CABG with CPB	Pro-MMP-9, Pro-MMP-2, TIMP-4	NOS	Changes of MMP-2, MMP-9, and NOS in human cardiac tissue during CABG	Pro-MMP-9 increased during CPB time in cardiac tissue and plasma. Pro-MMP-2 increased only in cardiac tissue. TIMP-4 decreased during CPB time. Positive correlation between tissue activity of pro-MMP-9 and tissue activity of NOS. Tissue activity of pro-MMP-9 increased with increased duration of CPB time.
Joofs, 2001 [12]	Prospective observational NRCT	28	Patients undergoing elective CABG with CPB	MMP-2, MMP-8, MMP-9, MMP-13	-	Plasmatic changes of MMP-2, MMP-8, MMP-9, MMP-13 during and after CPB	MMP-8 increased fourfold after CPB, returning to normal levels within 30 min after CPB. MMP-9 and MMP-13 increased more than twofold at cross-clamp release and returned to levels within normal limits within 6 h after CPB. MMP-2 increased from baseline values at 6 and 24 h post-operatively.
Galley, 2002 [13]	Prospective observational NRCT	20	Patients undergoing elective CABG with CPB	MMP-9, TIMP-1	TNF α	Relationship among MMP-9, TNF α, and TIMP-1 during CPB	MMP-9 increased during CPB. TIMP-1 decreased during CPB.
Lalu, 2005 [14]	Prospective observational NRCT	15	Patients undergoing elective CABG with CPB	MMP-2, MMP-9, TIMP-1	-	Changes in myocardial and plasma MMPs and TIMPs within 10 min of reperfusion and to determine whether these correlate with changes in acute post-ischaemic myocardial function	Increased MMP-2 and MMP-9 and decreased TIMP-1 in cardiac tissue. Increased MMP activity correlated positively with cross-clamp duration and inversely with cardiac function. TIMP-1 correlated inversely with cross-clamp time and positively with cardiac function. Increased plasma levels of MMP-2 and MMP-9 after declamping.
Lin, 2005 [15]	Prospective observational NRCT	21	Patients undergoing elective CABG with or without CPB	MMP-9, TIMP-1	Neutrophil	Correlation between MMP-9 and neutrophils during CPB	MMP-9 levels increased after beginning of CPB, whereas they did not increase in patients submitted to off-pump cardiac surgery. TIMP-1 increased gradually for 6 h. The MMP-9/TIMP-1 ratios increased 2–4 h after beginning of CPB.
Dorman, 2008 [7]	Prospective RCT	60	Patients undergoing elective CABG with CPB	MMP-2, MMP-8, MMP-9	IL-6, IL-10	Changes in ILs and MMPs in patients receiving aprotinin or epsilon-aminocaproic acid	MMP-2, MMP-8, and MMP-9 increased in the group receiving epsilon-aminocaproic acid after CPB. MMP-8 and MMP-9 remained elevated at 6 h.
Ng, 2008 [16]	Prospective RCT	50	Patients undergoing elective CABG with CPB	MMP-9, TIMP-1	IL-8, IL-10, TxB_2_	The effect of continuing ventilation during CPB on inflammatory reactions and cardiopulmonary function	MMP-9 levels were higher at 1, 4, and 6 h after declamping in the continuous ventilation group and in the non-ventilation group. TIMP-1 levels were higher in the continuous ventilation group.
Spinale, 2008 [17]	Prospective observational NRCT	14	Patients undergoing elective cardiac surgery with CPB	MMP-7, MMP-9	IL-6, TNFα	Changes in MMP activity in the myocardium after ischemia/reperfusion	MMP-9 and MMP-7 levels increased fourfold from the period of myocardial arrest to reperfusion.
Gong, 2011 [18]	Prospective RCT	30	Patients undergoing elective cardiac surgery with CPB	MMP-9	IL-6, TNFα	Changes in biomarkers of BPB-related inflammation after the inhalation of milrinone	MMP-9 increased at the end of surgery and 24 h after surgery in the overall population. After milrinone inhalation, MMP-9 levels were lower than the control levels at the end of surgery.
Zitta, 2014 [19]	Prospective RCT	35	Patients undergoing elective cardiac surgery with CPB	Pro-MMP-2, MMP-2, Pro-MMP-9, MMP-9	cardiac troponin T	MMP-2 and MMP-9 activity in remote ischemic preconditioning-mediated cardioprotection	After CPB, pro-MMP2, MMP-2, pro-MMP-9, and MMP-9 were not different in the overall population. Positive correlation between MMP-2 and MMP-9 and cardiac troponin T.
Lin, 2015 [20]	Prospective observational NRCT	30	Patients undergoing elective cardiac surgery with CPB	MMP-9	-	Changes in MMP-9 following CPB. To investigate the association between MMP-9 and PaO_2_/FiO_2_	MMP-9 levels increased 2, 4, and 6 h after CPB start, returning closely to the baseline at 24 h. MMP-9 levels at 4 and 6 h were not correlated with prolonged CPB time and PaO_2_/FiO_2_.
Beer, 2015 [21]	Prospective observational NRCT	30	Patients undergoing CABG with CPB	MMP-3, MMP-8, MMP-9	LCN-2	Difference in levels of MMP-3, MMP-8, MMP-9, TIMP-1, and LCN-2 with or without continuous mechanical ventilation during CPB	MMP-8, MMP-9, and LCN-2 were lower at the end of surgery in ventilated patients.
McNair, 2021 [22]	Prospective observational NRCT	30	Patients undergoing elective cardiac surgery with CPB	MMP-2, MMP-9	Serum creatinine	Changes in serum and urine MMP-2 and MMP-9 after CPB. To analyze MMP-2 and MMP-9 as early biomarkers of AKI	Serum and urine levels of MMP-2 and MMP-9 were higher in the AKI patients compared with non-AKI patients. MMP-2 and MMP-9 increased earlier than serum creatinine in AKI patients.
Fang, 2022 [23]	Retrospective observational NRCT	22	Patients undergoing pericardiectomy with CPB	MMP-1, MMP-2, MMP-9, TIMP-1	-	To evaluate the effect of MMPs and TIMPs on post-operative outcomes of patients with constrictive pericarditis undergoing pericardiectomy	MMP-9 was associated with postoperative complications, with an optimal cutoff predicting value of 3.67.
Gao, 2022 [6]	Prospective RCT	36	Patients undergoing elective CABG or valve replacement with CPB	MMP-2, MMP-9	Syndecan-1	Changes in MMPs with doxycycline administration	MMP-2 and MMP-9 levels were lower in the doxycycline group than those in the control group during CPB.

**Table 2:** Matrix metalloproteinases and tissue inhibitors of matrix metalloproteinases identified in human myocardium during and after cardiac surgery with cardiopulmonary bypass. (Abbreviations: MMP, matrix metalloproteinase; TIMP, tissue inhibitor of matrix metalloproteinase).

Subgroup	MMP	Nomenclature	Mass (kDa)	Substrate	References
Interstitial collagenase	MMP-1	Fibroblast collagenase	52	Collagens I, II, III, VI, VIII, and X, gelatin, aggrecan, MMP-2, MMP-9	[23]
	MMP-8	Neutrophil collagenase or collagenase 2	75	Collagens I, II, III, V, VII, VIII and X, gelatin, aggrecan	[7,12,21]
	MMP-13	Collagenase 3	54	Collagens I, II, III, and IV, gelatin, aggrecan	[12]
Gelatinases	MMP-2	Gelatinase A	72	Gelatin, collagen types I, IV, V, VII, X, XI, and XIV, elastin, fibronectin, aggrecan	[6,7,11,12,14,19,23]
	MMP-9	Gelatinase B	92	Gelatin, collagen types IV, V, VII, and X, elastin	[6,7,11,12,13,14,15,16,17,18,19,20,21,22,23]
Stromelysins	MMP-3	Stromelysin 1	57	Collagens III, IV, IX, and X, gelatin, aggrecan, MMP-1, MMP-7, MMP-8, MMP-9, MMP-13, laminin, fibronectin, non-helical collagen	[21]
	MMP-7	Matrilysin	28	Collagens IV and X, gelatin, fibronectin	[17]
Glycoproteins	TIMP-1	-	28	all MMPs except MMP-14	[13,14,15,16,23]
	TIMP-2	-	21	all MMPs	-
	TIMP-3	-	24	all MMPs	-
	TIMP-4	-	23	all MMPs	[11]

## Data Availability

All data generated or analyzed during this study are included in this published article.

## Supplementary Materials

The following supporting information can be downloaded at: https://www.mdpi.com/article/10.3390/biom13010113/s1, Figure S1: Preferred Reporting Items for Systematic Reviews and Meta-Analyses (PRISMA) flow diagram; Figure S2: Risk of bias graph: review authors’ judgements about each risk of bias item presented as percentages across all included studies; Figure S3: Risk of bias summary: review authors’ judgements about each risk of bias item for each randomized included study. Table S1: PICo criteria for inclusion and exclusion of studies; Table S2: Quality scoring for included papers using Newcastle–Ottawa Scale; Table S3: Characteristics of excluded studies. Commentary S1. Assessment of publication bias for v.

## References

[1] Squiccimarro E., Labriola C., Malvindi P.G., Margari V., Guida P., Visicchio G., Kounakis G., Favale A., Dambruoso P., Mastrototaro G. (2019). Prevalence and Clinical Impact of Systemic Inflammatory Reaction After Cardiac Surgery. J. Cardiothorac. Vasc. Anesthesia.

[2] Paparella D., Yau T., Young E. (2002). Cardiopulmonary bypass induced inflammation: Pathophysiology and treatment. An update. Eur. J. Cardio-Thorac. Surg..

[3] Cabral-Pacheco G.A., Garza-Veloz I., La Rosa C.C.-D., Ramirez-Acuña J.M., Perez-Romero B.A., Guerrero-Rodriguez J.F., Martinez-Avila N., Martinez-Fierro M.L. (2020). The Roles of Matrix Metalloproteinases and Their Inhibitors in Human Diseases. Int. J. Mol. Sci..

[4] Buchler A., Munch M., Farber G., Zhao X., Al-Haddad R., Farber E., Rotstein B.H. (2021). Selective Imaging of Matrix Metalloproteinase-13 to Detect Extracellular Matrix Remodeling in Atherosclerotic Lesions. Mol. Imaging Biol..

[5] DeLeon-Pennell K.Y., Meschiari C.A., Jung M., Lindsey M.L. (2017). Matrix Metalloproteinases in Myocardial Infarction and Heart Failure. Matrix metalloproteinases in myocardial infarction and heart failure. Prog. Mol. Biol. Transl. Sci..

[6] Gao W., Fang F., Xia T.J., Zhang Y., Sun J., Wu Q., Wang W. (2022). Doxycycline can reduce glycocalyx shedding by inhibiting matrix metalloproteinases in patients undergoing cardiopulmonary bypass: A randomized controlled trial. Microvasc. Res..

[7] Dorman B.H., Stroud R.E., Wyckoff M.M., Zellner J.L., Botta D., Leonardi A.H., Ikonomidis J.S., Spinale F.G. (2008). Differential Effects of Epsilon-aminocaproic Acid and Aprotinin on Matrix Metalloproteinase Release in Patients Following Cardiopulmonary Bypass. J. Cardiovasc. Pharmacol..

[8] Higgins J.P.T., Green S. (2011). Cochrane Handbook for Systematic Reviews of Interventions Version 5.1.0 [Updated March 2011]. The Cochrane Collaboration.

[9] Higgins J.P.T., Sterne J.A.C., Higgins J.P.T., Green S. (2011). Assessing risk of bias in included studies. Cochrane Handbook for Systematic Reviews of Interventions Version 5.1.0.

[10] Higgins J.P.T., Deeks J.J., Altman D.G., Higgins J.P.T., Green S. (2011). Chapter 16: Special topics in statistics. Cochrane Handbook for Systematic Reviews of Interventions Version 5.1.0.

[11] Mayers I., Hurst T., Puttagunta L., Radomski A., Mycyk T., Sawicki G., Johnson D., Radomski M.W. (2001). Cardiac surgery increases the activity of matrix metalloproteinases and nitric oxide synthase in human hearts. J. Thorac. Cardiovasc. Surg..

[12] Joffs C., Gunasinghe H.R., Multani M.M., Dorman B.H., Kratz J.M., Crumbley A.J., Crawford F.A., Spinale F.G. (2001). Cardiopulmonary bypass induces the synthesis and release of matrix metalloproteinases. Ann. Thorac. Surg..

[13] Galley H.F., Macaulay G.D., Webster N.R. (2002). Matrix metalloproteinase-9, tissue inhibitor of metalloproteinase-1 and tumour necrosis factor α release during cardiopulmonary bypass. Anaesthesia.

[14] Lalu M.M., Pasini E., Schulze C.J., Ferrari-Vivaldi M., Ferrari-Vivaldi G., Bachetti T., Schulz R. (2004). Ischaemia–reperfusion injury activates matrix metalloproteinases in the human heart. Eur. Heart J..

[15] Lin T.-C., Li C.-Y., Tsai C.-S., Ku C.-H., Wu C.-T., Wong C.-S., Ho S.-T. (2005). Neutrophil-Mediated Secretion and Activation of Matrix Metalloproteinase-9 During Cardiac Surgery with Cardiopulmonary Bypass. Anesthesia Analg..

[16] Ng C.S., Arifi A.A., Wan S., Ho A.M., Wan I.Y., Wong E.M., Yim A.P. (2008). Ventilation During Cardiopulmonary Bypass: Impact on Cytokine Response and Cardiopulmonary Function. Ann. Thorac. Surg..

[17] Spinale F.G., Koval C.N., Deschamps A.M., Stroud R.E., Ikonomidis J.S. (2008). Dynamic Changes in Matrix Metalloproteinase Activity Within the Human Myocardial Interstitium During Myocardial Arrest and Reperfusion. Circulation.

[18] Gong M., Lin X.-Z., Lu G.-T., Zheng L.-J. (2011). Preoperative Inhalation of Milrinone Attenuates Inflammation in Patients Undergoing Cardiac Surgery with Cardiopulmonary Bypass. Med. Princ. Pract..

[19] Zitta K., Meybohm P., Bein B., Gruenewald M., Lauer F., Steinfath M., Cremer J., Zacharowski K., Albrecht M. (2014). Activities of cardiac tissue matrix metalloproteinases 2 and 9 are reduced by remote ischemic preconditioning in cardiosurgical patients with cardiopulmonary bypass. J. Transl. Med..

[20] Lin T.-C., Lin F.-Y., Lin Y.-W., Hsu C.-H., Huang G.-S., Wu Z.-F., Tsai Y.-T., Lin C.-Y., Tsai C.-S. (2015). Matrix Metalloproteinase-9 Production following Cardiopulmonary Bypass Was Not Associated with Pulmonary Dysfunction after Cardiac Surgery. Mediat. Inflamm..

[21] Beer L., Warszawska J.M., Schenk P., Debreceni T., Dworschak M., Roth G.A., Szerafin T., Ankersmit H.J. (2014). Intraoperative ventilation strategy during cardiopulmonary bypass attenuates the release of matrix metalloproteinases and improves oxygenation. J. Surg. Res..

[22] McNair E.D., Bezaire J., Moser M., Mondal P., Conacher J., Franczak A., Sawicki G., Reid D., Khani-Hanjani A. (2021). The Association of Matrix Metalloproteinases With Acute Kidney Injury Following CPB-Supported Cardiac Surgery. Can. J. Kidney Health Dis..

[23] Fang L., Yu W., Yu G., Ye B., Chen G. (2022). Predictive value of matrix metalloprotease 9 on surgical outcomes after pericardiectomy. J. Cardiothorac. Surg..

[24] Carney D.E., Lutz C.J., Picone A.L., Gatto L.A., Ramamurthy N.S., Golub L.M., Simon S.R., Searles B., Paskanik A., Snyder K. (1999). Matrix Metalloproteinase Inhibitor Prevents Acute Lung Injury After Cardiopulmonary Bypass. Circulation.

[25] Guenzinger R., Lahm H., Wottke M., Lange R. (2012). Role of Metalloproteinases and Tissue Inhibitors of Metalloproteinases During Cardiopulmonary Bypass in Rats. ASAIO J..

[26] Zhang C., Gong W., Liu H., Guo Z., Ge S. (2014). Inhibition of matrix metalloproteinase-9 with low-dose doxycycline reduces acute lung injury induced by cardiopulmonary bypass. Int. J. Clin. Exp. Med..

[27] Wang C.-T., Zhang L., Wu H.-W., Wei L., Xu B., Li D.-M. (2014). Doxycycline attenuates acute lung injury following cardiopulmonary bypass: Involvement of matrix metalloproteinases. Int. J. Clin. Exp. Pathol..

[28] Irqsusi M., Mansouri A.L., Ramaswamy A., Rexin P., Salman M., Mahmood S., Mirow N., Ghazi T., Ramzan R., Rastan A.J. (2022). Role of matrix metalloproteinases in mitral valve regurgitation: Association between the of MMP-1, MMP-9, TIMP-1, and TIMP-2 expression, degree of mitral valve insufficiency, and pathologic etiology. J. Card. Surg..

[29] Sarkar M., Prabhu V. (2017). Basics of cardiopulmonary bypass. Indian J. Anaesth..

[30] Robich M., Ryzhov S., Kacer D., Palmeri M., Peterson S.M., Quinn R.D., Carter D., Sheppard F., Hayes T., Sawyer D.B. (2020). Prolonged Cardiopulmonary Bypass is Associated With Endothelial Glycocalyx Degradation. J. Surg. Res..

[31] Turner N.A., Porter K.E. (2012). Regulation of myocardial matrix metalloproteinase expression and activity by cardiac fibroblasts. IUBMB Life.

[32] Arpino V., Brock M., Gill S.E. (2015). The role of TIMPs in regulation of extracellular matrix proteolysis. Matrix Biol..

[33] Pardo A., Selman M. (2005). MMP-1: The elder of the family. Int. J. Biochem. Cell Biol..

[34] Phatharajaree W., Phrommintikul A., Chattipakorn N. (2007). Matrix metalloproteinases and myocardial infarction. Can. J. Cardiol..

[35] Momiyama Y., Ohmori R., Tanaka N., Kato R., Taniguchi H., Adachi T., Nakamura H., Ohsuzu F. (2010). High plasma levels of matrix metalloproteinase-8 in patients with unstable angina. Atherosclerosis.

[36] Quillard T., Tesmenitsky Y., Croce K., Travers R., Shvartz E., Koskinas K.C., Sukhova G.K., Aikawa E., Aikawa M., Libby P. (2011). Selective Inhibition of Matrix Metalloproteinase-13 Increases Collagen Content of Established Mouse Atherosclerosis. Arter. Thromb. Vasc. Biol..

[37] Gonçalves P.R., Nascimento L.D., Gerlach R.F., Rodrigues K.E., Prado A.F. (2022). Matrix Metalloproteinase 2 as a Pharmacological Target in Heart Failure. Pharmaceuticals.

[38] Huang X.-Y., Han L.-Y., Huang X.-D., Guan C.-H., Mao X.-L., Ye Z.-S. (2016). Association of Matrix Metalloproteinase-1 and Matrix Metalloproteinase-3 Gene Variants with Ischemic Stroke and Its Subtype. J. Stroke Cerebrovasc. Dis..

[39] Olejarz W., Łacheta D., Kubiak-Tomaszewska G. (2020). Matrix Metalloproteinases as Biomarkers of Atherosclerotic Plaque Instability. Int. J. Mol. Sci..

