# Supplementary material for: Metalloproteinases in Cardiac Surgery: A Systematic Review
Source: Biomolecules. 2023 Jan 5;13(1):113. doi: 10.3390/biom13010113 (PMC9855939; doi:10.3390/biom13010113)
Supplement: Supplementary file 1 [file biomolecules-13-00113-s001.zip › biomolecules-2080210-supplementary.pdf]

Supplementary Material

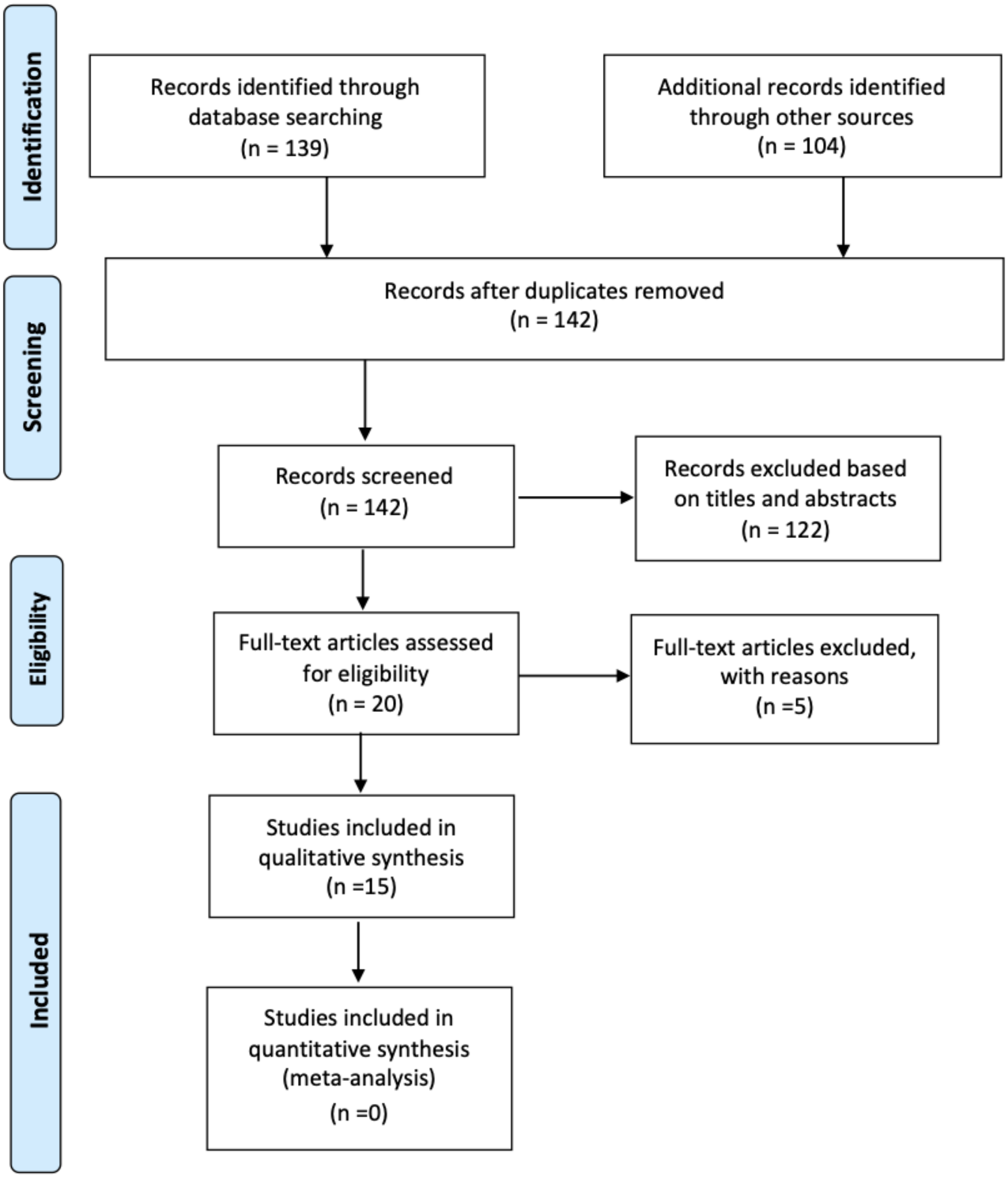

Figure S1. Preferred Reporting Items for Systematic Reviews and Meta-Analyses (PRISMA) flow diagram.

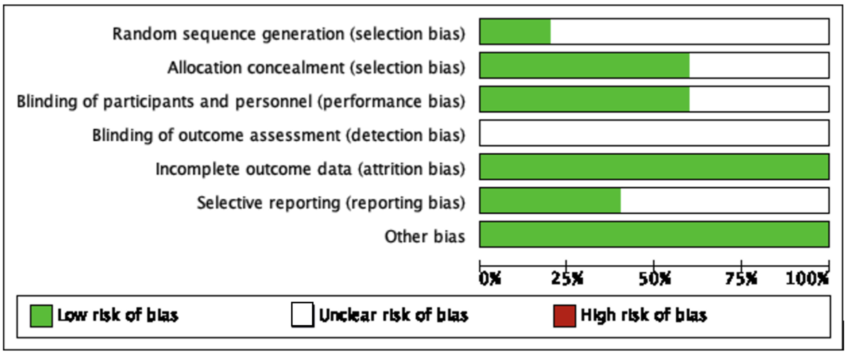

Figure S2. Risk of bias graph: review authors' judgements about each risk of bias item presented as percentages across all included studies.

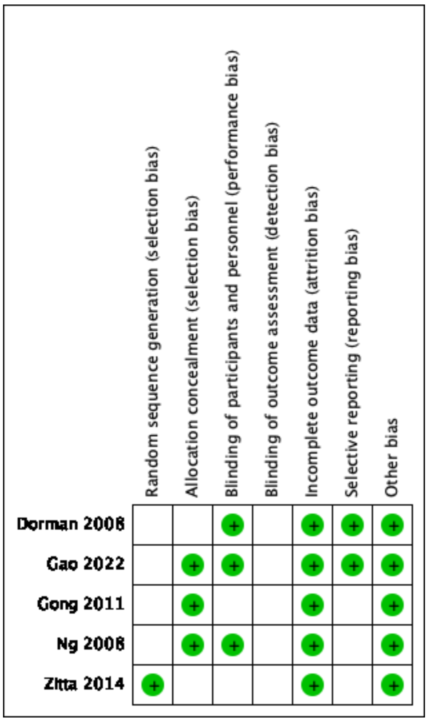

Figure S3. Risk of bias summary: review authors' judgements about each risk of bias item for each randomized included study.

Table S1. PICo criteria for inclusion and exclusion of studies

Parameter	Inclusion criteria	Exclusion criteria
Population/Patient/Problem	Patient undergoing cardiac surgery	-
Interest	Metalloproteinases analysis	-
Context	Cardiopulmonary bypass	-

Table S2. Quality scoring for included papers using Newcastle-Ottawa Scale

Study (Author, Year)	Selection	Comparability	Outcome	Exposure	*Total*
Mayers, 2001 [11]	3	-	1	–	*4*
Joffs, 2001 [12]	3	-	1	–	*4*
Galley, 2002 [13]	3	-	1	–	*4*
Lalu, 2005 [14]	3	-	1	–	*4*
Lin, 2005 [15]	4	1	1	–	*6*
Spinale, 2008 [17]	3	-	1	–	*4*
Lin, 2015 [20]	3	-	1	–	*4*
Beer, 2015 [21]	4	1	1	–	*6*
McNair, 2021 [22]	4	1	1	–	*6*
Fang, 2022 [23]	3	-	1	–	*4*

A study can be awarded a maximum of 4 points for the Selection category, 2 points for the comparability category and 3 points for the Outcome/Exposure categories. Therefore, the maximum points a study can obtain is 9 which indicates a high-quality study.

Table S3. Characteristics of excluded studies

Study, year [Ref]	Type of study	N° of patients	Population	Metalloproteinase	Outcomes	Main findings	Reason for exclusion
Carney, 1999 [24]	Prospective observational NRCT	26	Yorkshire pigs	MMP-2	To analyze the effect of MMP inhibitor, a chemically modified tetracycline, on acute lung injury prevention	Prevention of lung dysfunction followed an attenuation of both elastase and MMP-2 activity.	Animal model
Guenzinger, 2012 [25]	Prospective observational NRCT	80	Wistar rats	MMP-2, MMP-9, TIMP-1, TIMP-4	To evaluate the role of MMPs after CPB	Increased MMPs levels	Animal model
Wang, 2014 [27]	Prospective observational RCT	48	Sprague-Dawley rats	MMP-9	The effect of doxycycline on MMPs activity	Decreased concentration and activity of MMP-9	Animal model
Zhang, 2014 [26]	Prospective observational RCT	30	Mongrel dogs	MMP-9	The effect of doxycycline on MMPs activity	Decreased concentration and activity of MMP-9	Animal model
Irqsusi, 2022 [28]	Prospective observational NRCT	80	Patient undergoing elective mitral valve	MMP-1, MMP-9, TIMP-1, TIMP-2	To analyze a relationship between MMPs	An increased expression of MMPs and TIMPs was	In vitro analysis.

			surgery with CPB		and mitral regurgitation	observed in mild mitral insufficiency, decreasing with the increase of mitral insufficiency severity	CPB role was not investigated.

Commentary S1. Assessment of publication bias for RCT

Dorman, 2008 [7]

Domain	Support for judgement	Risk of bias
**Random sequence generation (selection bias)**	Not stated.	Unclear
**Allocation concealment (selection bias)**	Not stated.	Unclear
**Blinding of participants and personnel (performance bias)**	The surgeon was blinded to the randomization protocol, but due to the differences in dosing regimens, the anaesthesiologist was not.	Low
**Blinding of outcome assessment (detection bias)**	Not stated.	Unclear
**Incomplete outcome data (attrition bias)**	Complete outcome data	Low
**Selective reporting (reporting bias)**	This work was supported by National Heart, Lung, and Blood Institute Grants PO1-HL-48788, RO1-HL-59165, RO1- HL56603 and a Merit Review Award from the Department of Veterans Affairs.	Low
**Other bias**	Appears to be free of other bias	Low

Ng, 2008 [16]

Domain	Support for judgement	Risk of bias
**Random sequence generation (selection bias)**	Not stated.	Unclear
**Allocation concealment (selection bias)**	Prior to surgery, a dedicated researcher randomized patients by opening a presealed envelope to the continuous ventilation or the nonventilated groups during CPB.	Low
**Blinding of participants and personnel (performance bias)**	Except for the anesthetists and surgeon, the laboratory technicians and other clinicians caring for the patients were blinded to the intraoperative ventilation status.	Low
**Blinding of outcome assessment (detection bias)**	Not stated.	Unclear
**Incomplete outcome data (attrition bias)**	Complete outcome data	Low

**Selective reporting (reporting bias)**	Not stated.	Unclear
**Other bias**	Appears to be free of other bias	Low

Gong, 2011 [18]

**Domain**	**Support for judgement**	**Risk of bias**
**Random sequence generation (selection bias)**	Not stated.	Unclear
**Allocation concealment (selection bias)**	Patients were randomly divided into two groups by using sealed envelopes.	Low
**Blinding of participants and personnel (performance bias)**	Not stated.	Unclear
**Blinding of outcome assessment (detection bias)**	Not stated.	Unclear
**Incomplete outcome data (attrition bias)**	Complete outcome data	Low
**Selective reporting (reporting bias)**	Not stated.	Unclear
**Other bias**	Appears to be free of other bias	Low

Zitta, 2014 [19]

**Domain**	**Support for judgement**	**Risk of bias**
**Random sequence generation (selection bias)**	Patients were randomized to group RIPC or control in a double-blinded fashion.	Low
**Allocation concealment (selection bias)**	Not stated.	Unclear
**Blinding of participants and personnel (performance bias)**	Not stated.	Unclear
**Blinding of outcome assessment (detection bias)**	Not stated.	Unclear
**Incomplete outcome data (attrition bias)**	Complete outcome data	Low
**Selective reporting (reporting bias)**	Not stated.	Unclear
**Other bias**	Appears to be free of other bias	Low

Gao, 2022 [6]

Domain	Support for judgement	Risk of bias
**Random sequence generation (selection bias)**	Not stated.	Unclear
**Allocation concealment (selection bias)**	The randomization sequence, generated by Microsoft Office Excel, was sealed in opaque envelopes to conceal the group assignments.	Low
**Blinding of participants and personnel (performance bias)**	The randomization and allocation were blinded to the investigators and patients, except for the staff who enrolled the patients, generated randomization, and prepared drugs. This staff was not involved with the care of any enrolled patients or the assessment of the outcomes.	Low
**Blinding of outcome assessment (detection bias)**	Not stated.	Unclear
**Incomplete outcome data (attrition bias)**	Complete outcome data	Low
**Selective reporting (reporting bias)**	Trial reported. Selective reporting found [Zhejiang Provincial National Natural Science Foundation of China (LY20H090008). Wenzhou Science and Technology Bureau (Y2020162).)]	Low
**Other bias**	Appears to be free of other bias	Low
